# Causal Relationship Between Gluten-Free Diet and Autoimmune-Related Disease Risk: A Comprehensive Mendelian Randomization Study

**DOI:** 10.7150/ijms.104928

**Published:** 2025-01-01

**Authors:** Yi Peng, Chenxi Liu, Wei Liu, Runxin Gan

**Affiliations:** 1 Department of Rheumatology and Immunology, Xiangya Hospital, Central South University, Changsha, Hunan, 410008, China.; 2 Department of Cardiology, Shanghai East Hospital, School of Medicine, Tongji University, Shanghai, 200120, China.; 3 Department of Hematology, Xiangya Hospital, Central South University, Changsha, Hunan, 410008, China.; 4 Reproductive Medicine Center, Xiangya Hospital, Central South University, Changsha, Hunan, 410008, China.; 5 National Clinical Research Center for Geriatric Disorders, Xiangya Hospital, Changsha, Hunan, 410008, China.

**Keywords:** gluten-free diet, autoimmune-related diseases, rheumatoid arthritis, immune cells, cytokine

## Abstract

While the gluten-free diet (GFD) is primarily used to treat celiac disease (CD), recent research suggests it may also offer benefits for autoimmune-related diseases (ARDs), though findings remain inconsistent. This study aimed to investigate the potential protective effect of a GFD against ARDs by Mendelian Randomization (MR) analysis. Utilizing data from over 500,000 samples from the UK Biobank and other publicly available genome-wide association studies (GWAS), MR analysis revealed a significant negative causal relationship between GFD and the risk of developing rheumatoid arthritis (RA) (OR = 0.782, 95% CI = [0.727-0.841], p < 0.001). Mediation analysis identified immune cells such as CD14+ CD16+ monocyte absolute count (mediating 2.441% of the effect), CD14+ CD16+ monocyte percentage (2.346%), and CD20 on IgD+ CD38^dim B cells (3.119%) as potential mediators in the protective effect of GFD on RA. These findings suggest that GFD may help reduce RA risk by modulating specific immune cell populations. However, further research is necessary to clarify the exact mechanisms underlying these associations.

## Introduction

A gluten-free diet (GFD) involves strictly avoiding gluten-containing foods (e.g., wheat, barley, rye) and using gluten-free options as the main carbohydrate source (e.g., rice, millet, corn, buckwheat). Initially developed for treating celiac disease (CD), a strict GFD is currently the only effective treatment, alleviating symptoms, promoting mucosal healing, and preventing complications^1^. Recently, an increasing number of non-CD individuals have adopted the GFD, with reports indicating that 72% of new GFD adopters in the U.S. are people without celiac disease avoiding gluten (PWAG)^2^. Additionally, the GFD is gaining popularity in the Asia-Pacific region, including China, Japan, and India, as a perceived healthier option. Research predicts that the gluten-free food and beverage market in this region will grow at a compound annual growth rate (CAGR) of 9.34% from 2024 to 2029^3^. However, it is crucial to note that no official guidelines currently support GFD benefits for non-CD patients, and existing studies on its effects on certain autoimmune-related diseases (ARDs) show varying conclusions and insufficient evidence.

ARDs encompass various conditions marked by the loss of self-tolerance and the production of autoantibodies. Additionally, conditions where autoimmune dysfunction is recognized as a key pathogenic mechanism are included. Some research suggests that a GFD may benefit conditions such as psoriasis, rheumatoid arthritis (RA), and inflammatory bowel disease; however, conclusions from related studies are inconsistent, and the scarcity of large-scale trials renders the available evidence unreliable.

Mendelian randomization (MR) is a recent research approach that utilizes genetic variation as an instrumental variable (IV) to determine whether risk factors causally influence health outcomes. This method is advantageous when randomized controlled trials are impractical or when confounding factors cannot be eliminated in observational studies^4^. This study aimed to estimate the potential risk of ARDs associated with GFD exposure using MR analysis, intending to provide new evidence for the use of GFD.

## Materials and Methods

### Study design

**Figure 1:**
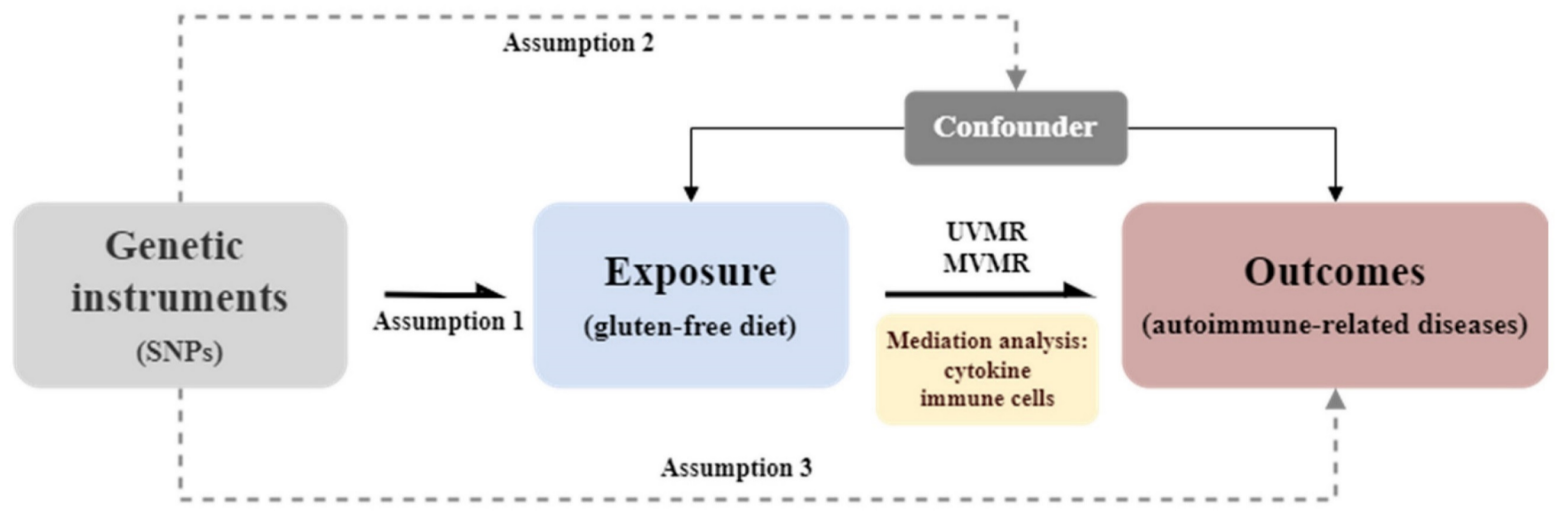
** Study design flowchart.** Assumption 1: The genetic variants used as instrumental variables are reliably associated with the exposure (i.e., gluten-free diet). Assumption 2: The instrumental variables are not associated with any confounding factors. Assumption 3: The instrumental variables influence the outcome (i.e., autoimmune-related diseases) solely through the exposure and not through any other direct causal pathways. UVMR, univariable mendelian randomization; MVMR, multivariable mendelian randomization.

The study design flowchart is presented in Figure 1, with all procedures conducted following STROBE-MR guidelines^5^. This MR study included six ARDs from various GWAS datasets as outcomes. Initial associations between GFD and these diseases were estimated using UVMR, leading to the selection of RA for further analysis, as GFD was identified as a potential protective factor.

Subsequently, various cytokines and immune cells were selected as potential mediators for mediation effect analysis. The MR mediation framework evaluated whether these mediators influenced the associations identified by UVMR. In this model, mediators were treated as outcomes of GFD and exposure factors for RA, with ORs calculated for each. Mediating effects were further assessed using comprehensive MR analysis. When sufficient instrumental variables are available for MVMR, MR mediation analysis is applied; otherwise, a two-step mediation approach is used.

The genomic data utilized in this study were sourced from publicly available databases and previously published GWAS. All relevant data received approval from the respective institutional ethics review committees, and informed consent was obtained from all participants.

### IVs screening

The SNPs used as instrumental variables were sourced from the UK Biobank (https://www.ukbiobank.ac.uk/), with additional calculations, including r² values, performed using the 1000 Genomes super populations^6^ as a reference. To eliminate linkage disequilibrium (LD), SNPs with a p-value > 5 × 10⁻⁸, r² > 0.001, and a clump distance < 10,000 kb were excluded from the analysis^7^. The same screening criteria were applied in the MR mediation analysis. Further details are provided in** Table 1.**

### Data source of ARDs

The classification of diseases in this study adheres to the International Classification of Diseases-10 (ICD-10) standard^8^, with relevant SNP data sourced from various publicly available GWAS. Detailed information on the involved GWAS is presented in **Table 1.**

### Statistical analysis

The SNPs associated with GFD, ARDs, and relevant exposure factors (such as immune cells) were harmonized based on ethnicity, chromosomal location, and SNP codes. Before analysis, *F*-statistics for all IVs were verified to be greater than 10 to exclude weak IVs. To mitigate the effects of missing data, the clump data function in the *TwoSampleMR* R package was utilized, and missing IVs were substituted with proxy SNPs from the National Center for Biotechnology Information (NCBI).

Inverse variance weighted (IVW) analysis was the primary method used in this study, incorporating both IVW (multiplicative random effects) and IVW (fixed effects) to assess the causal relationship between GFD exposure and ARDs occurrence. Additional methods included MR Egger^9^, Weighted median^10^, Simple mode, Weighted mode, and Maximum-Likelihood^11^ as supplementary analyses. Depending on the availability of sufficient IVs, mediation analysis utilized adjusted results from MVMR or two-step MR to estimate mediation effects for positive associations identified in UVMR. The Sobel, Aroian, and Goodman tests, along with the mediation^12^ and Rmediation R-packages^13^ were employed to calculate mediation effects, standard errors (SE), and 95% confidence intervals (CI).

Sensitivity analyses consisted of three components: assessing heterogeneity, evaluating horizontal pleiotropy, and performing a leave-one-out analysis. *Cochran's Q* statistic was calculated using the *MR-Egger*^9^ method to evaluate heterogeneity among the IVs. In cases of detected heterogeneity, a *random-effects IVW* method was employed; otherwise, a *fixed-effects IVW* method was used^14^. Horizontal pleiotropy was assessed via the *p*-value from the *MR-Egger* regression intercept test, with *p* < 0.05 indicating a significant association.

All analyses in this study were conducted using the R software (version 4.0.5) utilizing the *TwoSampleMR*^15^, *MR-PRESSO*^16^, and *MendelianRandomization*^17^ R-packages.

## Results

Before inclusion in the analysis, *F*-statistics for all IVs were ensured to be greater than 10 to exclude weak IVs. The final three SNPs were identified as strong IVs and included in the analyses. Details of all included IVs are presented in **Table 2**.

### Causal effects of GFD on autoimmune-related diseases

**Figure 2:**
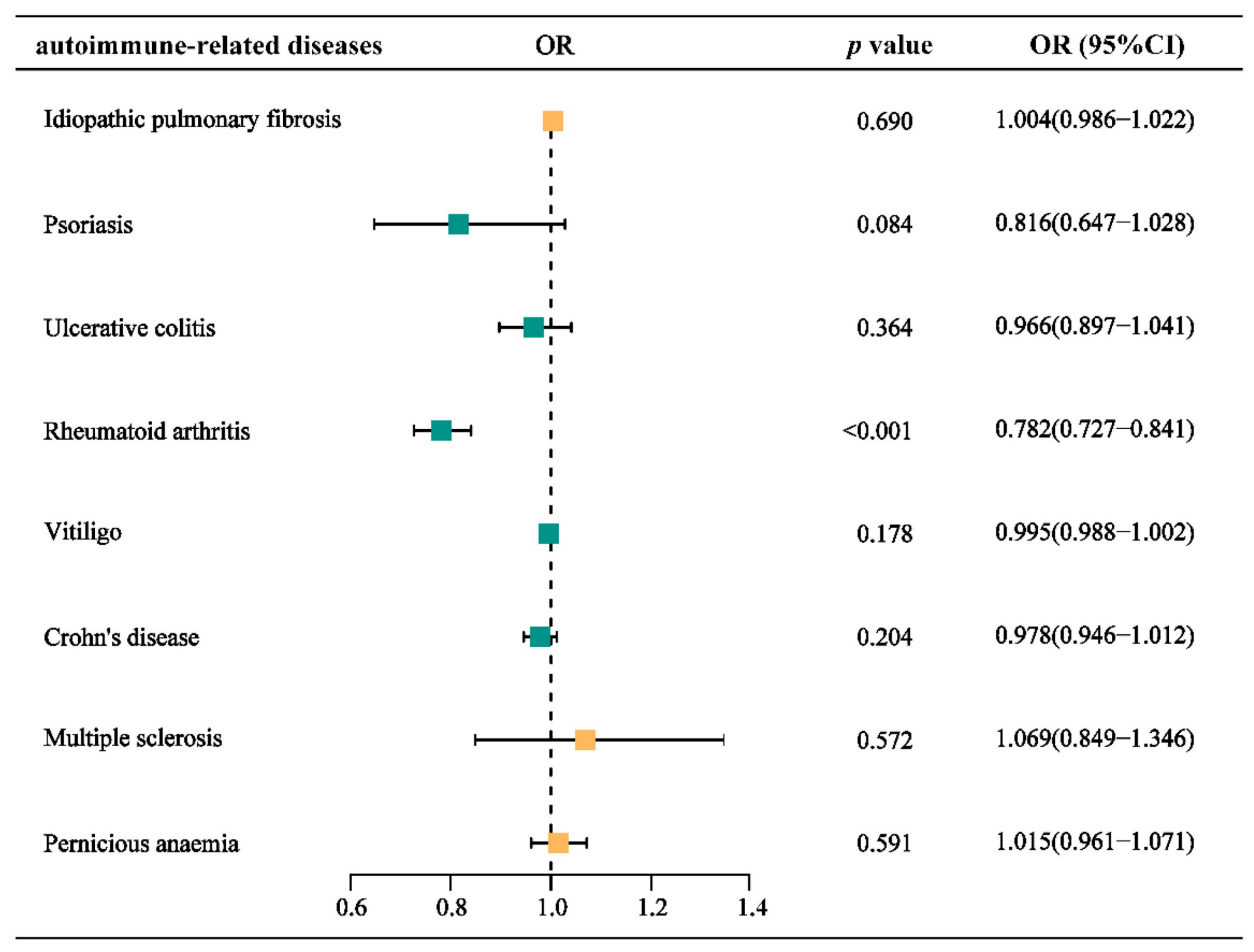
** Forest plot for the causal effects of gluten-free diet on the risk of different autoimmune-related diseases.** OR, odds ratio.

After analyzing the correlation between a GFD and the risk of developing several ARDs, we found that a GFD was significantly associated with RA and could serve as a protective factor (OR = 0.782, 95% CI = [0.727-0.841], p <0.001). The relevant results are presented as a forest plot in **Figure 2**, with detailed information provided in the **Supplementary Table ST-1**.

We conducted a sensitivity analysis to assess the reliability of the MR results. Heterogeneity was evaluated using the *MR-Egger* method, and no significant heterogeneity was detected between GFD and each autoimmune-related disease (all *p*-values > 0.05). Additionally, the *MR-Egger* intercept test indicated no clear evidence of horizontal pleiotropy in the data (all p-values > 0.05)** (Table 3, Supplementary Table ST-2)**.

### Two-step mediation analysis of GFD, RA, and immune cells

**Figure 3:**
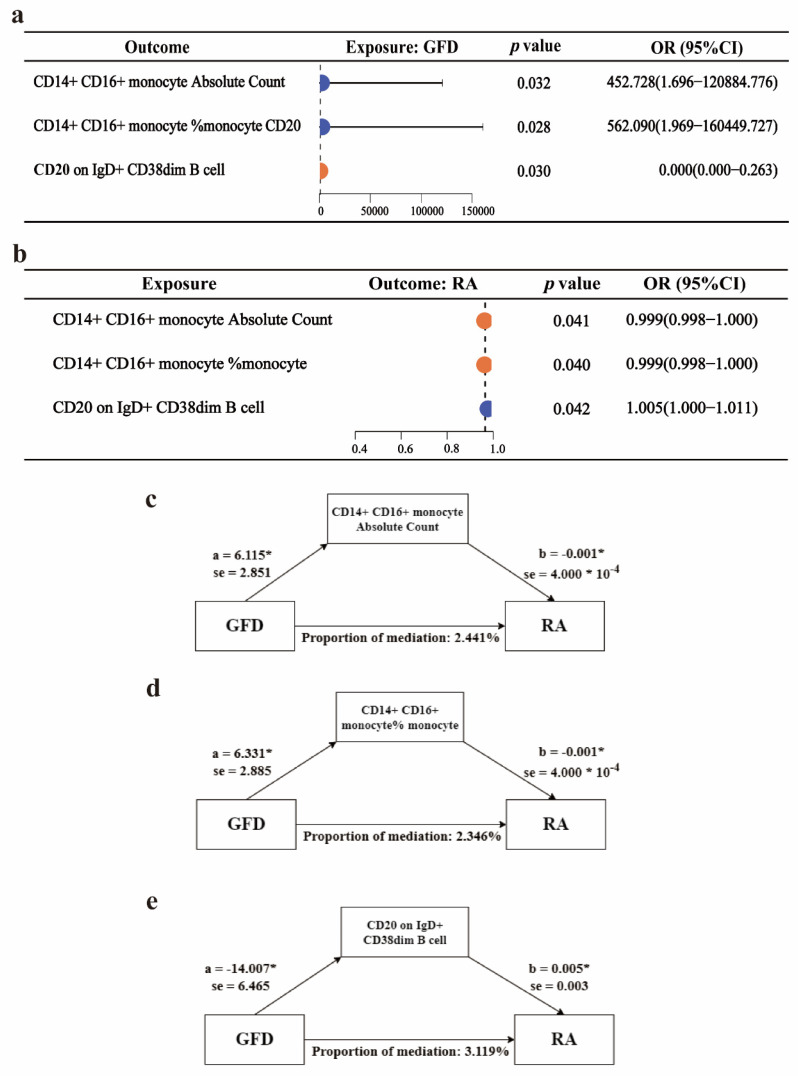
** Two-step mediation analysis of GFD, rheumatoid arthritis, and immune cells.** a. Forest plot for the causal effects of GFD on three types of immune cells. b. Forest plot for the causal effects of three types of immune cells on RA risk. c-d. Mediating effects of different immune cells. OR, odds ratio; GFD, gluten-free diet; RA, rheumatoid arthritis.

To explore possible mediators of GFD amelioration of RA, we introduced cytokines of RA progression for mediation analysis. Due to the insufficient number of IVs, we employed a two-step mediation analysis to estimate the associations between GFD and immune cells, as well as between immune cells and RA **(Figure 3a-b)**. Additionally, we further assessed the mediating role of immune cells in these associations. The results indicated that CD14+ CD16+ monocyte absolute count, CD14+ CD16+ monocyte %monocyte (The proportion of CD14+ CD16+ monocytes among monocytes), and CD20 on IgD+ CD38^dim B cells (CD20 expression level on IgD+ CD38^dim B cells) may act as mediators of the protective effect of a GFD on RA. Specifically, CD14+ CD16+ monocyte absolute count mediated 2.441% of the effect, CD14+ CD16+ monocyte %monocyte mediated 2.346% of the effect, and CD20 on IgD+ CD38^dim B cells mediated 3.119% of the effect **(Figure 3c-e, Supplementary Table ST-3)**.

### MVMR analysis of GFD, RA, and circulating cytokine levels

**Figure 4:**
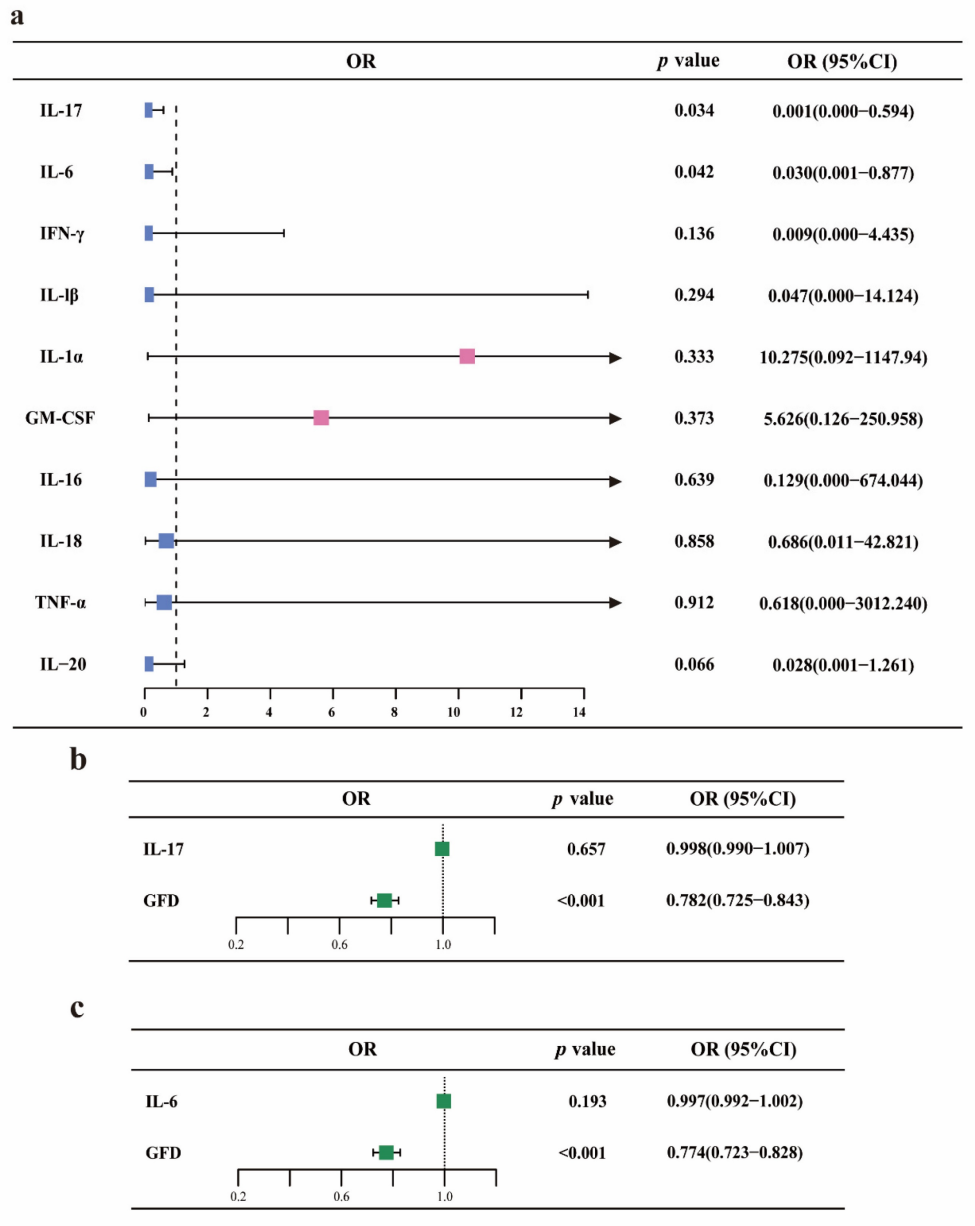
** MVMR analysis of GFD, rheumatoid arthritis, and circulating cytokine levels.** a. Forest plot for the causal effects of GFD on the levels of ten different cytokines. b-c. Forest plot showing the MVMR analysis results for GFD, IL-6/IL-17, and RA. OR, odds ratio; GFD, gluten-free diet; RA, rheumatoid arthritis.

Furthermore, we introduced cytokines involved in RA progression for MVMR analysis. Initially, MR analysis using the *IVW* or *Wald ratio* method indicated that GFD appears to have a protective effect on IL-17 and IL-6 levels. However, there was insufficient evidence to suggest that GFD affects the levels of IFN-γ, IL-1β, IL-1α, GM-CSF, IL-16, IL-18, TNF-α, and IL-20. MVMR analysis of IL-17 and IL-6 as mediators showed that GFD does not exert its protective effect against RA by altering these two cytokines, suggesting that the underlying mechanism may need further investigation. The relevant results are presented as a forest plot in **Figure 4**, with detailed information provided in **Supplementary Table ST-4**.

## Discussion

In this study, we utilized genome-wide data from public databases and selected genetic instruments to investigate the potential relationships between GFD and the risk of various ARDs. Initially, UVMR was employed to evaluate the impact of GFD exposure on the risk of eight ARDs. We identified a potential protective effect of GFD against RA, prompting further analyses that focused primarily on this relationship. Through MR mediation analysis, we found that GFD may confer protection against RA by modulating CD14+ CD16+ monocyte absolute count, CD14+ CD16+ monocyte %monocyte, and CD20 on IgD+ CD38^dim B cells. Although UVMR suggested that GFD reduces IL-6 and IL-17 levels, MVMR-adjusted results indicated that these cytokines do not mediate the protective effect of GFD on RA.

Previous studies have suggested that GFD may influence immune function by modulating intestinal permeability. Under normal physiological conditions, the gut maintains mucosal tolerance, ensuring that antigen transport across the mucosa is strictly controlled. Gliadin, a component of gluten, is resistant to human protease digestion, and its presence in the gut can trigger the upregulation of zonulin. This, in turn, disrupts tight junctions and increases intestinal permeability, potentially contributing to immune dysregulation^18^-^20^. Although this effect is more pronounced in individuals with CD, it can also occur in healthy individuals. This mechanism supports the belief that a GFD may benefit immune regulation. Some clinical studies have investigated whether GFD exposure can improve conditions related to autoimmunity or inflammation. A German prospective intervention study demonstrated that a short-term GFD had anti-inflammatory effects, evidenced by a reduction in peripheral blood leukocyte count, C-reactive protein (CRP) levels, and plasma inflammatory markers. However, the study found no significant improvement in cardiovascular disease outcomes^21^. Several studies have evaluated the role of GFD in improving fibromyalgia^22^-^25^, indicating that GFD may benefit a subset of patients with increased intraepithelial lymphocytosis. Similarly, research on GFD and autoimmune thyroiditissuggests that GFD may benefit women with chronic autoimmune thyroiditis (CAT) but normal thyroid function, as it was found to reduce serum antibody titers in these patients^26^-^29^. However, GFD does not appear to have a significant impact on TSH and FT4 levels^29^. Some studies have also explored the effects of GFD on RA, suggesting that its protective effect on RA is limited. However, it is crucial to account for the influence of confounding factors in the design of these studies, such as the patients' ethnicity, gender, age, and medication use. Moreover, in many of these studies, a GFD is often accompanied by vegetarianism, and evidence regarding the isolated effect of GFD on RA remains limited^30^-^32^. In this study, we applied MR, a method that effectively reduces confounding bias. Our findings suggest that GFD may have a protective effect against RA among various ARDs.

RA is an autoimmune disease that primarily affects the joints and surrounding soft tissues. A hallmark of RA is the production of autoantibodies and the involvement of autoreactive immune cells^33^. Therefore, we introduced immune cells and inflammatory factors as mediators in a mediation MR analysis to explore the potential mechanisms through which GFD may exert a protective effect against RA. Using two-step MR analysis, we found that GFD may positively affect CD14+ CD16+ monocyte absolute count and CD14+ CD16+ monocyte %monocyte, while negatively influencing CD20 on IgD+ CD38^dim B cells. Additionally, CD14+ CD16+ monocyte absolute count and CD14+ CD16+ monocyte %monocyte may reduce the risk of RA, whereas CD20 on IgD+ CD38^dim B cells may increase it. (Notably, the relevant immune cell entries are interpreted solely as phenotypes, and the OR does not reflect the absolute levels). These findings suggest that these immune cells could mediate the protective effect of GFD against RA. While tissue-invasive T effector cells have been key players in RA pathogenesis, the role of monocyte-macrophages in RA has recently gained increasing attention.^34^, ^35^. CD14+CD16+ monocytes, also known as intermediate monocytes, are capable of producing pro-inflammatory cytokines such as TNF-α, IL-1β, and IL-6. These cytokines promote inflammation, playing a significant role in inflammatory responses^36^, ^37^. Studies have demonstrated that CD14+ CD16+ monocytes are significantly elevated in RA patients compared to healthy individuals, with the highest levels observed in newly diagnosed cases. These monocyte levels positively correlate with disease severity, indicating their potential role in disease progression. Moreover, after treatment with biological therapies, RA patients often exhibit a decrease in CD14+ CD16+ monocyte levels, suggesting that these therapies may help modulate immune responses by reducing the pro-inflammatory monocyte population^38^, ^39^. Another study similarly identified significantly elevated levels of CD14+ CD16+ monocytes in the synovial fluid of RA patients, indicating their potential central role in shaping the inflammatory microenvironment within the joints^37^. These findings offer mechanistic support for the conclusions drawn from our MR analysis. Notably, existing therapies, particularly monoclonal antibodies targeting CD20, are well-established in improving RA outcomes, this may explain why a GFD can protect against RA by acting on IgD+ CD38^dim B cells.

Cytokine-driven biological processes are also central to the pathogenesis of RA. These cytokines act as critical mediators, driving immune cell differentiation, inflammation, and tissue pathology, thus playing a key role in disease progression. In this study, UVMR analysis suggested that GFD may potentially reduce the levels of IL-6 and IL-17. IL-6 is a pleiotropic cytokine that promotes the proliferation and differentiation of various cells, including the induction of naïve CD4+ T cells into Th17 cells in synergy with TGF^40^-^42^. In RA, IL-6 plays a critical role in synovial inflammation, osteoclast-mediated bone destruction, and pannus formation^43^, ^44^. IL-17, a pro-inflammatory cytokine primarily secreted by Th17 cells, promotes the activation of fibroblast-like synoviocytes (FLS) in RA patients and, along with IL-6, drives osteoclastogenesis. Furthermore, IL-17 mediates the recruitment of macrophages and neutrophils, amplifying the inflammatory response^45^-^47^. In a prospective study on GFD treatment for primary sclerosing cholangitis and associated colitis, it was noted that while improvements in the disease itself were limited, GFD reduced serum inflammatory cytokine levels, including IL-6^48^. This supports the findings of our MR analysis. However, after MVMR correction, IL-6 and IL-17 were not found to mediate the protective effect of GFD on RA, potentially due to limitations in the IVs and the quality of the dataset. Additionally, it is important to recognize the complexity of GFD effects; some studies have demonstrated that GFD can alter gut microbiota, which may be one of the mechanisms by which it protects against RA^49^, ^50^.

Despite the implementation of rigorous design and process controls, this study has several limitations. Firstly, the limited availability of IVs may introduce model-fitting bias, potentially affecting the reliability of the analysis results. Secondly, since all relevant data were sourced from public databases, it is impossible to determine whether there is overlap between individuals across different cohorts or to account for other confounding factors. Importantly, the genetic instruments utilized in this study are derived from individuals of European ancestry, which may limit the applicability of the results to other populations. Additionally, this study focuses solely on the impact of GFD on the risk of ARDs in healthy individuals; further investigation is needed to ascertain whether similar benefits apply to patients with ARDs. In conclusion, this study, utilizing MR analysis, suggests a potential protective effect of a GFD on the risk of RA, providing strong supplementary evidence to previous observational studies or clinical trials. However, further clinical trials focusing on patients from different populations and with varying disease activity states are essential to validate the findings of this study.

## Conclusions

In this study, MR analysis indicated that a GFD may protect against RA, with CD14+ CD16+ monocyte absolute count, CD14+ CD16+ monocyte %monocyte, and CD20 on IgD+ CD38^dim B cells identified as potential mediating factors.

## Supplementary Material

Supplementary tables.

## Figures and Tables

**Table 1:** Details of the GWAS data

Traits	IVs	Sample size	GWAS ID/PMID	Consortium
Exposure				
GFD	3	64,949 (1,376 cases/63,573control)	ukb-b-11189	MRC-IEU
Outcome				
Idiopathic pulmonary fibrosis	NA	451,025 (1,369 cases/435,866 control)	33197388	EBI database
Psoriasis	NA	484,598 (5,427 cases/479,171 control)	33959723	EBI database
Ulcerative colitis	NA	484,598 (2,515 cases/482,083 control)	33959723	EBI database
Rheumatoid arthritis	NA	484,598 (5,427 cases/479,171 control)	33959723	UK Biobank
vitiligo	NA	337,159 (95 cases/337,064 control)	ukb-a-115	UK Biobank
Crohn's disease	NA	461,460 (732 cases/336,467 control)	ukb-a-552	MRC-IEU
multiple sclerosis	NA	115,803 (47,429 cases/68,374 control)	ieu-b-18	MRC-IEU
pernicious anaemia	NA	462,933 (1,401 cases/461,532 control)	ukb-b-8720	EBI database

**Table 2:** Included instrumental variables of the gluten-free diet

SNPs	Chr	Beta	Se	*p*-val	Related Genes
rs1548306	6	-0.007	0.001	0.001	NA
rs9271842	6	0.006	0.001	<0.001	NA
rs9273595	6	0.010	0.001	0.002	HLA-DQB1

**Table 3:** Results of sensitivity analysis

Outcomes	*Q* statistic	*p* for *Q* statistic	Egger intercept	*p* for intercept
Idiopathic pulmonary fibrosis	0.040	0.842	<0.001	0.868
Psoriasis	2.399	0.121	-0.006	0.103
Ulcerative colitis	0.166	0.683	0.002	0.140
Rheumatoid arthritis	3.178	0.0747	-0.001	0.402
Multiple sclerosis	270.193	<0.001	<0.001	0.971
Pernicious anemia	6.124	0.013	-0.001	0.398
